# Amino acid digestibility and protein quality of fermented soybean-based ingredients using the precision-fed cecectomized rooster assay

**DOI:** 10.1093/jas/skaf328

**Published:** 2025-09-20

**Authors:** Meredith A Smola, Pamela L Utterback, Carl M Parsons, Xin Chen, Zhenjia Chen, Yan Liu, Perry K W Ng, Kelly S Swanson

**Affiliations:** Department of Animal Sciences, University of Illinois Urbana-Champaign, Urbana, IL 61801; Department of Animal Sciences, University of Illinois Urbana-Champaign, Urbana, IL 61801; Department of Animal Sciences, University of Illinois Urbana-Champaign, Urbana, IL 61801; Department of Food Science and Human Nutrition, Michigan State University, East Lansing, MI 48824; Department of Food Science and Human Nutrition, Michigan State University, East Lansing, MI 48824; Department of Biosystems and Agricultural Engineering, Michigan State University, East Lansing, MI 48824; Department of Food Science and Human Nutrition, Michigan State University, East Lansing, MI 48824; Department of Animal Sciences, University of Illinois Urbana-Champaign, Urbana, IL 61801; Division of Nutritional Sciences, University of Illinois Urbana-Champaign, Urbana, IL 61801

**Keywords:** canine nutrition, feline nutrition, fermented soybeans, nutrient digestion, pet food

## Abstract

Soybean meals, soy protein isolates, and other forms of soy have served as protein sources in pet foods for many years. The amino acid (AA) content and protein quality of soybean-based ingredients vary depending on their composition and processing, however, so the testing of new ingredients is required. Our objective was to measure the AA composition, AA digestibility, and protein quality of fermented soybean-based ingredients using the precision-fed cecectomized rooster assay. Cecectomized roosters were randomly allotted to one of five test ingredients (n = 6/ingredient): 1) autoclaved soybeans (ASB); 2) fermented soybeans (FSB); 3) fermented soybeans + *Lactococcus lactis subsp. lactis* ATCC 11454 (FSBP); 4) fermented soybean meal (FSBM); and 5) fermented soybean meal + *L. lactis* (FSBMP). After 26 h of feed withdrawal, roosters were tube-fed test ingredients. Following crop intubation, excreta samples were collected for 48 h. Endogenous losses were accounted for by using 5 additional fasted cecectomized roosters. In addition to calculating AA digestibility, digestible indispensable AA score (DIAAS)-like values were determined and first limiting AA were identified based on dog and cat nutrient requirements or recommendations from the National Research Council (NRC). All data were analyzed by the Mixed Models procedure in SAS version 9.4. All ingredients had acceptable AA digestibilities, with all indispensable AA digestibilities being >80%, with the exception of histidine (79.3%), lysine (73.5%), and valine (79.0%) for ASB. All AA digestibility values were different (*P *< 0.05) among ingredients, with FSBP usually being the most digestible and ASB usually being the least digestible. The DIAAS-like values were highest for FSBP. The criteria for a high-quality protein source for adult cats was met by FSB, FSBP, FSBM, and FSBMP, with ASB meeting the criteria for a good-quality protein. The DIAAS-like values based on NRC recommendations for adult dogs, growing puppies, and growing kittens were lower. For adult dogs, growing dogs, and growing kittens, methionine + cystine was the first limiting AA. For adult cats, phenylalanine + tyrosine was first limiting. Differences existed, but our results show that fermented soybean-based ingredients have moderately high AA digestibilities and may serve as adequate proteins in pet foods.

## Teaser

This study was conducted to measure the amino acid composition, amino acid digestibility, and protein quality of fermented soybean-based ingredients using the precision-fed cecectomized rooster assay. The results demonstrated that all ingredients had high amino acid digestibilities and suggest that they may serve as adequate protein sources in dog and cat foods.

## Introduction

Soybeans hold a prominent position as an oilseed worldwide. According to the Food and Agriculture Organization, worldwide soybean production is approximately 420.6 million metric tons (United States Department of Agriculture (USDA) Foreign Agricultural Services, 2025). Soybeans provide a rich source of amino acids (AA), polyunsaturated fatty acids, and dietary fiber (Zhang and Laflamme, 1999). However, they also contain anti-nutritional factors such as trypsin inhibitors, allergens, and nondigestible oligosaccharides (stachyose, raffinose, and verbascose) that may negatively affect nutrient availability and animal health and limit their use in pet food (Stein et al., 2008). Processing technologies may remove or reduce soybean anti-nutritional factors, thereby increasing AA digestibility and protein quality and allowing greater use by the pet food industry.

Current pet food trends are increasing opportunities for utilization of soybeans and other plant-based proteins. In recent years, the pet food industry has shifted more of its focus towards functional health benefits and sustainability, as the market continues to mature (Kim et al., 2023a). In the United States, there are approximately 20 million pet owners who follow a vegetarian lifestyle. In a recent survey, 54% of all pet owners expressed interest in providing a plant-based diet for their pets regardless of their dietary preferences (Dodd et al., 2019). Vegan pet food formulated with plant-based proteins is also a growing trend among pet owners (Nicholles and Knight, 2025). The advantages of incorporating soybeans into pet food formulations include their nutritional value, palatability, and functional processing properties. There are a multitude of soybean-based ingredients, all with different chemical and physical characteristics. This variety enables formulators to use the ingredients as a means to adjust dietary nutrient digestibility, fiber fermentability, and expansion of the final product (Kim et al., 2023a). Fermentation may be used to deactivate trypsin inhibitors, remove anti-nutritional factors (for example, oligosaccharides), and increase AA and crude protein (CP) concentrations (Cervantes-Pahm and Stein, 2010). A variety of fermentation methods may be used to process feed ingredients, but often includes the use of bacteria (for example, *Bacillus*, *Lactococcus*), filamentous fungi (for example, *Aspergillus oryzae*), or microbial combinations (Prado et al., 2022). Those processes are different than biomass fermentation using microorganisms to generate a protein-rich biomass (for example, mycoprotein) and precision fermentation using genetically modified microorganisms (for example, *Saccharomyces cerevisiae*) to produce ingredients and dietary components.

Fermented soybean-based ingredients with *Lactococcus lactis subsp. lactis* ATCC 11454 in a deactivated or activated form may be used as a protein source in pet food. *L. lactis* has a proteolytic system that hydrolyzes soybean proteins into peptides, uptakes these peptides into the cell, and degrades these peptides to free AA. Given these actions, improved AA digestibility and potential improvements to protein quality would be expected in soybeans fermented by *L. lactis*. By utilizing the nitrogen source of soybean-based ingredients, *L. Lactis* produces nisin, the antimicrobial peptide that aids in food preservation (Liu et al., 2017). Because each ingredient’s composition, fermentation process, and activity of *L. lactis*, contributes to a unique AA profile and protein quality of soybean-based ingredients, however, testing is required.

The cecectomized rooster assay is a common model used to measure nutrient and AA digestibility of individual feed ingredients (for example, insect proteins, cultured bacterial proteins, cultured yeast proteins, novel animal-based proteins) or complete and balanced pet foods (Do et al., 2021; Oba et al., 2023; Smola et al., 2023) because the results have been shown to be similar to ileal-cannulated dogs (Johnson et al., 1998). Similar to ileal-cannulated dogs, cecectomized roosters allow for the AA digestibility estimates with minimal interference from the bacterial fermentation of proteins in the hindgut. Additionally, the cecectomized rooster assay is more affordable, more time efficient and less labor intensive than ileal-cannulated dogs (Johnson et al., 1998; Faber et al., 2010). Thus, the cecectomized rooster assay is often the preferred model to evaluate the protein quality of novel ingredients for dogs and cats (Oba et al., 2019).

The objective of the current study was to measure the AA composition, AA digestibilities, and protein quality of soybean-based ingredients intended for use in pet foods using the precision-fed cecectomized rooster assay. We hypothesized that all ingredients would have moderately high AA digestibilities, but higher in soybeans fermented by *L. lactis*. We also hypothesized that the digestible indispensable amino acid score (DIAAS)-like values would differ among ingredients based on ingredient variation and processing methods.

## Materials and Methods

The protocol for the cecectomized rooster assay, including all animal housing, handling, and surgical procedures, was reviewed and approved by the Institutional Animal Care and Use Committee at the University of Illinois Urbana-Champaign prior to experimentation (IACUC #23092).

### Ingredients

Five soybean-based ingredients were tested: 1) autoclaved soybeans (ASB); 2) fermented soybeans (FSB); 3) fermented soybeans + *Lactococcus lactis subsp. lactis* ATCC 11454 (FSBP); 4) fermented soybean meal (FSBM); and 5) fermented soybean meal + *L. lactis* (FSBMP). All ingredients (500 g) were mixed with 1.5 liter of water in beakers. Beakers were double sealed with aluminum foil and autoclaved at 121 °C for 15 min for sterilization using a Thermo Scientific ST75925 Sterilemax Benchtop Steam Sterilizer (Thermo Fisher Scientific, MA). The autoclaving process was repeated at 121 °C for 15 min. For the ASB ingredient, soybeans were autoclaved and then dried at 65 °C and served as the control. The other ingredients were fermented by *L. lactis* after being autoclaved. *Lactococcus lactis subsp. lactis* ATCC 11454 was activated using yeast peptone dextrose agar plates and cultured at 35 °C for 48 h. Single colonies were inoculated into fresh yeast peptone dextrose broth and cultured at 35 °C for 24 h. 20 mL of the activated *L. lactis* broth was inoculated into 200 mL yeast peptone dextrose medium and cultured using the same conditions and used as the seed for soybean and soybean meal fermentations. Soybean or soybean meal ingredients (500 g) and 200 mL of the activated *L. lactis* were added to and mixed with the sterilized soybean and soybean meal medium. Fermentations were carried out at 35 °C for 24 h. The FSB and FSBM ingredients were dried at 65 °C to deactivate the *L. lactis*, whereas the FSBP and FSBMP ingredients were dried at 37 °C to keep the *L. lactis* alive. All dried ingredients were ground using a Thomas-Wiley laboratory mill (Thermo Fisher Scientific, MA) to obtain a particle size less than 2 mm.

### Cecectomized rooster assay

A precision-fed rooster assay using cecectomized Single Comb White Leghorn roosters was conducted as described by Parsons (1985) to determine the AA digestibility of the ingredients listed above. Prior to the study, the cecectomy surgery was performed on roosters under general anesthesia according to the procedures of Parsons (1985). All roosters were given at least 8 wk to recover from surgery before being used in experiments.

Briefly, 30 cecectomized roosters (n = 6/group) were randomly assigned to one of five test ingredients using a random assignment generator in Excel. After 26 h of feed withdrawal, roosters were tube-fed (crop intubation) 25 g of test ingredient. Following crop intubation, excreta were collected for 48 h on plastic trays placed under each individual cage. Excreta samples then were lyophilized, weighed, and ground through a 0.25-mm screen prior to analyses. Endogenous loss corrections for AA were made by using 5 additional cecectomized roosters that had been fasted for 26 h followed by an additional fasting period of 48 h during which excreta were collected quantitatively, lyophilized, and analyzed for AA to estimate endogenous losses. Amino acid digestibilities were calculated using the method described by Engster et al. (1985). All birds were housed individually in cages (27.9 cm wide × 50.8 cm long × 53.3 cm high) with raised wire floors. They were kept in an environmentally controlled room (approximately 23.9 °C, 17 h light:7 h dark). Before the start of the experiment, feed and water were supplied ad libitum.

### Chemical analyses

The ingredients and rooster excreta were analyzed for dry matter (DM; 105 °C) and ash according to Association of Official Analytical Chemists (AOAC) (2006) with organic matter (OM) being calculated (DM: method 934.01; ash: method 942.05). Crude protein of the ingredients was determined by Leco Nitrogen/Protein Determinator (TruMac N, Leco Corporation, St Joseph, MI) total nitrogen values according to Association of Official Analytical Chemists (AOAC) (2006; method 992.15). Amino acids were measured at the University of Missouri Experimental Station Chemical Laboratories (Columbia, MO) according to Association of Official Analytical Chemists (AOAC) (2006; method 982.30E). Total lipid content was determined using acid hydrolysis and extraction methods facilitated by ANKOM Technology equipment (Hydrolysis System, XT15 Extractor, and RD Dryer; Macedon, NY). Ingredient total dietary fiber was determined according to Prosky et al. (1992). Trypsin inhibitor concentrations were analyzed using method Ba 12–75 (American Oil Chemists Society (AOCS), 2006). Concentrations of the oligosaccharides, stachyose, raffinose, and verbascose, were analyzed using high-preformace liquid chromatography according to Smiricky et al. (2002). Briefly, ingredients were homogenized with water, placed in an 80 °C water bath, and incubated for 1 h. The incubation was followed by centrifugation utilizing a Centriprep with a 10,000 molecular weight cutoff, and the filtrate was used for chromatographic analysis. Eluted oligosaccharides were quantified using a Dionex (DX-500) high-preformace liquid chromatography system consisting of an AS 50 autosampler, a GP 50 gradient pump module, and a pulsed electrochemical detector, equipped with a gold working electrode. Each assay was run in duplicate.

### Amino acid digestibility calculations

Basal endogenous AA losses were determined using roosters that were fasted for 48 h and then standardized AA digestibility values were calculated by the method of Engster et al. (1985) using the equation below.


AA digestibility of diet (%) = [(AA consumed—AA excreted by fed birds + AA excreted by fasted birds)/AA consumed]× 100

where AA consumed (g) = diet intake (g) × AA in diet (%); AA excreted by fed birds (g) = excreta output (g) × AA in excreta (%); AA excreted by fasted birds = excreta output (g) × AA in excreta (%).

### DIAAS-like calculations

The calculation of DIAAS-like values was preformed according to Mathai et al. (2017) and Oba et al. (2019). The digestible indispensable AA reference ratio was calculated for each ingredient using the following equation (Food and Agriculture Organization (FAO), 2013):


Digestible indispensable AA reference ratio = mg digestible indispensable AA content in 1 g protein of food/mg of the same dietary indispensable AA in 1 g of the reference protein.

The references used were the National Research Council (National Research Council (NRC), 2006) minimal requirements for growing puppies (4 to 14 wk of age) and growing kittens and the NRC recommended allowances for adults (dogs and cats), growing puppies (4 to 14 wk of age), and growing kittens. The DIAAS-like values were then calculated using the following equation adapted from (Food and Agriculture Organization (FAO), 2013):


Digestible indispensable AA reference ratio = [(mg digestible indispensable AA/1 g test ingredient CP)/(mg same indispensable AA/1 g CP requirement)]× 100

This equation produced a reference ratio for each indispensable AA, with the lowest value representing the first or most limiting AA of each protein source. In accordance with the Food and Agriculture Organization (FAO) (2013), DIAAS-like thresholds were used to distinguish ingredients considered to be “excellent” (DIAAS ≥ 100) or “good” (DIAAS from 75 to 99) quality proteins from those not carrying a protein quality claim (DIAAS < 75).

### Statistical analyses

All data were analyzed using the Mixed Models procedure of SAS (v. 9.4; SAS Institute Inc, Cary, NC). Ingredients were tested and considered to be fixed effects, with roosters being considered random effects. Differences among ingredients were determined using a Fisher-protected least significant difference with a Tukey adjustment to control for experiment-wise error. Normality of residuals was checked using the univariate procedure and Shapiro-Wilk statistic, with log transformation being used when normal distribution was lacking. Differences were considered statistically significant with *P *< 0.05.

## Results

### Chemical composition

The analyzed chemical composition of the fermented soybean-based ingredients tested in this study is presented in Table 1. The OM and fat concentrations were highest for FSB (94.64% OM and 20.13% fat on DM basis) and lowest for FSBMP (92.53% OM and 0.50% fat on DM basis). Crude protein was highest for FSBMP (50.41% CP, DM basis) and lowest for ASB (43.64% CP, DM basis). Total dietary fiber was highest for FSB (37.69%, DM basis) and lowest for ASB (22.58%, DM basis). The concentrations of oligosaccharides raffinose and stachyose were highest in FSBMP (8.86 mg/g raffinose, DM basis; 46.52 mg/g stachyose, DM basis) and lowest in FSB (4.00 mg/g raffinose, DM basis; 25.37 mg/g stachyose, DM basis). The concentration of another oligosaccharide, verbascose, was highest in ASB (1.91 mg/g, DM basis) and lowest in FSBM (1.23 mg/g, DM basis). The concentrations of trypsin inhibitor for FSBM, FSBMP, FSBP, FSB, and ASB were 0.80, 0.97, 2.50, 3.04, and 5.55 units/mg, respectively. Concentrations of indispensable and dispensable AA are presented in Table 1, with concentrations of most tending to be similar relative to CP (highest concentrations in FSBMP; lowest concentrations in ASB).

**Table 1.** Analyzed chemical composition of fermented soybean-based ingredients

Item	ASB^1^	FSB	FSBP	FSBM	FSBMP
Dry matter	96.56	95.34	94.77	95.86	93.21
	- % dry matter basis -				
Organic matter	94.49	94.64	94.44	92.87	92.53
Ash	5.51	5.36	5.56	7.13	7.47
Crude protein	43.64	44.34	44.05	50.19	50.41
Acid-hydrolyzed fat	19.30	20.13	18.70	0.71	0.50
Total dietary fiber	22.58	37.69	34.76	26.81	34.21
Oligosaccharides					
Raffinose, mg/g	4.87	4.00	5.30	8.42	8.86
Stachyose, mg/g	30.46	25.37	28.98	44.96	46.52
Verbascose, mg/g	1.91	1.78	1.79	1.23	1.24
Trypsin inhibitor, TIU/mg	5.55	3.04	2.50	0.80	0.97
Indispensable AA					
Arginine	3.44	3.27	3.18	3.56	3.66
Histidine	1.21	1.30	1.30	1.47	1.51
Isoleucine	2.23	2.28	2.28	2.65	2.75
Leucine	3.50	3.56	3.55	4.19	4.33
Lysine	2.61	2.88	2.89	3.30	3.51
Methionine	0.58	0.59	0.58	0.69	0.71
Phenylalanine	2.35	2.42	2.40	2.80	2.89
Threonine	1.72	1.73	1.72	1.99	2.09
Tryptophan	0.45	0.45	0.58	0.66	0.70
Valine	2.32	2.37	2.34	2.76	2.88
Selected dispensable AA					
Alanine	1.97	2.00	1.93	2.33	2.40
Aspartic acid	5.15	5.21	5.19	5.94	6.15
Cysteine	0.60	0.64	0.60	0.69	0.64
Glutamic acid	8.43	8.21	8.14	9.51	9.68
Proline	2.24	2.29	2.19	2.57	2.63
Serine	2.07	1.88	1.90	2.13	2.20
Tyrosine	1.58	1.69	1.67	1.84	1.92

1 ASB, autoclaved soybeans; FSB, fermented soybeans; FSBP, fermented soybeans + *Lactococcus lactis subsp. lactis* ATCC 11454; FSBM, fermented soybean meal; FSBMP, fermented soybeans + *L. lactis*; TIU, trypsin inhibitor units.

### Amino acid digestibilities

Amino acid digestibilities of fermented soybean-based ingredients are presented in Table 2. All ingredients had moderately high AA digestibilities, with all indispensable AA digestibilities being >80% with the exception of histidine (79.3%), lysine (73.5%), and valine (79.0%) for ASB. Arginine digestibility was higher (*P *< 0.05) in FSBP than ASB and FSB, and higher (*P *< 0.05) in FSBM than ASB. Histidine digestibility was higher (*P *< 0.05) in FSBP and FSBM than ASB. Isoleucine digestibility was higher (*P *< 0.05) in FSBP than ASB, FSB, and FSBMP, and higher (*P *< 0.05) in FSBM than ASB. Leucine digestibility was higher (*P *< 0.05) in FSBP than ASB and FSBMP, and higher (*P *< 0.05) in FSBM than ASB. Lysine digestibility was lower (*P *< 0.05) in ASB than the other ingredients. Methionine digestibility was higher (*P *< 0.05) in FSBP than ASB and FSBMP, and higher (*P *< 0.05) in FSB and FSBM than ASB. Phenylalanine digestibility was higher (*P *< 0.05) in FSBP than ASB, FSB, and FSBMP, and higher (*P *< 0.05) in FSBM than ASB. Threonine digestibility was higher (*P *< 0.05) in FSBP than ASB and FSBMP. Tryptophan digestibility was lower (*P *< 0.05) in ASB and FSB than the other ingredients. Valine digestibility was higher (*P *< 0.05) in FSBP than ASB, FSB, and FSBMP, and higher (*P *< 0.05) in FSBM than ASB. Alanine digestibility was higher (*P *< 0.05) in FSBP than ASB and FSBMP, and higher (*P *< 0.05) in FSBM than ASB. Aspartic acid digestibility was lower (*P *< 0.05) in FSBMP than the other ingredients. Cysteine, glutamic acid, and proline digestibilities were higher (*P *< 0.05) in FSBP than ASB and FSBMP. Serine digestibility was higher (*P *< 0.05) in FSBP than ASB, FSB, and FSBMP. Tyrosine digestibility was higher (*P *< 0.05) in FSBP than ASB, FSB, and FSBMP, and higher (*P *< 0.05) in FSB and FSBM than ASB.

**Table 2.** Amino acid (AA) digestibility (%) of soybean-based ingredients differing in processing method using the precision-fed cecectomized rooster assay

Item	ASB^1^	FSB	FSBP	FSBM	FSBMP	SEM	P-value
Indispensable AA							
Arginine	87.9^c^	89.2^bc^	93.1^a^	91.7^ab^	90.0^abc^	0.85	0.0018
Histidine	79.3^b^	82.8^ab^	86.3^a^	85.1^a^	82.1^ab^	1.22	0.0042
Isoleucine	81.8^c^	85.1^bc^	89.2^a^	87.4^ab^	85.2^bc^	0.90	<0.0001
Leucine	82.5^c^	85.7^bc^	90.1^a^	88.2^ab^	86.0^bc^	0.89	<0.0001
Lysine	73.5^b^	83.6^a^	86.9^a^	84.2^a^	83.1^a^	1.32	<0.0001
Methionine	81.5^c^	85.8^ab^	88.3^a^	87.0^ab^	84.7^bc^	0.81	<0.0001
Phenylalanine	83.8^c^	87.1^bc^	90.7^a^	89.3^ab^	87.3^bc^	0.83	<0.0001
Threonine	81.0^b^	83.6^ab^	87.5^a^	83.8^ab^	81.6^b^	1.12	0.0039
Tryptophan	88.6^b^	88.8^b^	95.1^a^	92.5^a^	91.7^a^	0.74	<0.0001
Valine	79.0^c^	82.9^bc^	87.3^a^	84.6^ab^	82.8^bc^	1.00	0.0001
Selected dispensable AA							
Alanine	78.2^c^	81.9^abc^	85.0^a^	82.7^ab^	80.2^bc^	0.96	0.0006
Aspartic acid	81.1^a^	84.1^a^	83.1^a^	79.1^a^	73.7^b^	1.27	<0.0001
Cysteine	68.4^b^	74.7^ab^	77.3^a^	72.9^ab^	68.8^b^	1.78	0.0058
Glutamic acid	86.2^b^	88.4^ab^	91.3^a^	89.7^ab^	87.6^b^	0.84	0.0027
Proline	84.4^b^	87.5^ab^	91.5^a^	88.9^ab^	86.8^b^	1.00	<0.0001
Serine	83.4^b^	85.2^b^	89.4^a^	85.7^ab^	84.4^b^	0.92	0.0014
Tyrosine	85.3^c^	89.2^b^	93.1^a^	90.5^ab^	88.7^bc^	0.88	<0.0001

1 ASB, autoclaved soybeans; FSB, fermented soybeans; FSBP, fermented soybeans + *Lactococcus lactis subsp. lactis* ATCC 11454; FSBM, fermented soybean meal; FSBMP, fermented soybeans + *L. lactis*.

a–d Within a row, means lacking a common superscript differ (*P *< 0.05); *n *= 6 roosters per ingredient.

### DIAAS-like values for dogs and cats

The DIAAS-like values representing the limiting AA of the test ingredients based on the NRC requirements and recommendations for dogs are presented in Table 3. According to the NRC requirements and recommendations for adult dogs at maintenance and growing puppies, methionine + cystine was the first limiting AA for all test ingredients. Based on the Food and Agriculture Organization (FAO) (2013), the test ingredients did not reach the threshold for a good protein source (>75).

**Table 3.** Digestible indispensable AA scores (DIAAS)-like values^1^ and associated first limiting AA with the lowest score of fermented soybean-based ingredients as determined using National Research Council (NRC) requirements and recommendations for dogs

Ingredient	Adult maintenance		Early growth^1^	
ASB^2^	30.29	MET+CYS	63.30	MET+CYS
FSB	33.23	MET+CYS	69.32	MET+CYS
FSBP	33.43	MET+CYS	69.85	MET+CYS
FSBM	32.74	MET+CYS	68.41	MET+CYS
FSBMP	30.02	MET+CYS	62.73	MET+CYS

1 Early growth = 4 to 14 wk of age.

2 ASB, autoclaved soybeans; FSB, fermented soybeans; FSBP, fermented soybeans + *Lactococcus lactis subsp. lactis* ATCC 11454; FSBM, fermented soybean meal; FSBMP, fermented soybeans + *L. lactis*.

The DIAAS-like values representing the first limiting AA of the test ingredients based on NRC requirements and recommendations for cats are presented in Table 4. According to the NRC requirements and recommendations for adult cats at maintenance, phenylalanine + tyrosine was the first limiting AA for all ingredients. When using the NRC requirements and recommendations for growing kittens, methionine + cystine was the first limiting AA for all ingredients. Based on the Food and Agriculture Organization (FAO) (2013), all ingredients met the standard for an excellent protein source (≥100) when using the NRC requirements and recommendations for adult cats at maintenance, with the exception of ASB that met the standard for a good protein source (75 to 99) according to the NRC for adult cats at maintenance. The test ingredients did not reach the threshold for a good protein source (>75) when using the NRC requirements and recommendations for growing kittens.

**Table 4.** Digestible indispensable AA scores (DIAAS)-like values and associated first limiting AA with the lowest score of fermented soybean-based ingredients as determined using National Research Council (NRC) requirements and recommendations for cats

Ingredient	Adult maintenance		Growing	
ASB^1^	96.85	PHE+TYR	50.63	MET+CYS
FSB	103.89	PHE+TYR	55.53	MET+CYS
FSBP	108.25	PHE+TYR	55.87	MET+CYS
FSBM	105.12	PHE+TYR	54.73	MET+CYS
FSBMP	103.28	PHE+TYR	50.18	MET+CYS

1 ASB, autoclaved soybeans; FSB, fermented soybeans; FSBP, fermented soybeans + *Lactococcus lactis subsp. lactis* ATCC 11454; FSBM, fermented soybean meal; FSBMP, fermented soybeans + *L. lactis*.

## Discussion

As the world population and consumption of food continues to increase, alternative proteins are needed, as traditional animal-based protein ingredients such as poultry, pork, and beef may become scarce (Food and Agriculture Organization (FAO), 2009). Furthermore, environmental challenges surround livestock production, such as pasture degradation, soil erosion, reduction in biodiversity, disruption of water cycles, and the emission of greenhouse gases (Swanson et al., 2013). To ensure that human and pet food systems continue to deliver nutritious diets while preserving a healthy ecosystem for future generations, it is essential to identify and/or develop sustainable protein sources. Increasing the amount of protein produced is only part of the solution, as ingredients differ in nutrient concentrations and digestibility. Evaluating the AA concentrations, AA digestibility, and protein quality of ingredients is crucial to ensure that all AA requirements will be met (Engster et al., 1985). The current study used the DIAAS-like procedure using cecectomized roosters to estimate protein quality of ingredients and diets for dogs and cats, which is similar to the DIAAS procedure that is used to estimate protein quality of ingredients and diets for humans (Mathai et al., 2017). The models vary slightly (cecectomized rooster vs. ileal-cannulated pig) and the “reference protein” for the DIAAS-like procedure uses the NRC nutrient requirements or recommendations, but the equations used were similar to those defined by the Food and Agriculture Organization (FAO) (2013) and have been used in several recent studies (Oba et al., 2019; Do et al., 2020; Oba et al., 2020; Do et al., 2021; Smola et al., 2023).

A multitude of soybean-based ingredients (that is, soybean meal; enzyme-treated soybean meal; soy protein isolate; soy protein concentrate; hydrolyzed soybean protein) are used in pet foods. Soy is a major oilseed in the United States, offering not only a rich source of polyunsaturated fatty acids, but also protein and fermentable fiber (Kim et al., 2023a). Soybean-based products typically serve as complementary protein sources in primarily grain-based pet diets because the AA profile of soybean products is not complete (Hill, 2003). Nevertheless, previous studies have demonstrated its value as a protein source in companion animals. For example, a study conducted by Clapper et al. (2001) evaluated soybean-containing diets in ileal-cannulated dogs. Results showed that neither ileal nor total tract macronutrient digestibility was affected by soy protein processing. There were no significant differences among treatments in apparent ileal digestibilities of DM, OM, fat, or gross energy. Soybean-containing diets had similar CP digestibilities (average = 85.1%). Apparent ileal AA digestibility data showed that arginine, histidine, isoleucine, leucine, lysine, phenylalanine, and valine digestibilities were higher (*P *< 0.01 and *P *< 0.05) for soy protein-containing diets and soy protein concentrate-containing diets than those for poultry meal. Murry et al. (1997) evaluated the ileal and total tract macronutrient digestibility of 5 isonitrogenous dry dog foods containing defatted soy flour, rendered beef meat and bone meal, fresh beef, poultry by-product meal, and fresh poultry. Results showed no differences among treatments in ileal digestibilities of DM, OM, CP, fat, or gross energy. Bednar et al. (2000) confirmed these results in a study evaluating soybean meal compared with beef meat and bone meal, soy flour, 3 soy protein concentrates, and poultry by-product meal.

In a canine study conducted by Kim et al. (2023b) evaluating the apparent total tract macronutrient digestibility and palatability of extruded kibble diets with 0%, 10%, 20%, or 30% whole soybeans, differences were noted. In that study, linear increases (*P* < 0.05) in fecal moisture, output, and defecation frequency were shown with increasing whole soybean inclusion levels. Likewise, linear decreases (*P *< 0.05) in apparent total tract macronutrient digestibly of DM, OM, CP, fat, and gross energy were observed with increasing whole soybean inclusion levels. Fecal total short-chain fatty acid, acetate, and propionate concentrations increased linearly (*P *< 0.05), while total branch-chained fatty acid concentrations decreased linearly (*P *< 0.05) with increasing whole soybean inclusion levels. The greater short-chain fatty acid concentrations are considered a positive outcome because they contribute to intestinal and host health by serving as energy substrates for colonic epithelial cells, maintaining epithelial barrier, regulating energy metabolism, and providing anti-inflammatory effects (Swanson and Fahey, 2006; Yang and Wu, 2023). Collectively, these studies demonstrate that soybean-based ingredients may serve as an alternative to traditional animal protein by-products in pet food and may have positive benefits to host health if used at proper inclusion levels.

Fermented soybean-based ingredients have been evaluated in weanling pigs (Cervantes-Pahm and Stein, 2010). Results showed that the apparent and standardized ileal AA digestibilities were not different between fermented soybean meal and soybean meal, with the exception of lysine being lower (*P *< 0.05) in fermented soybean meal, and that standardized ileal digestibilies of most AA were similar to that of fish meal. Few studies have tested the use of fermented soybean-based ingredients as a protein source for pets, however. The fermented soybean-based ingredients evaluated in the current study differed in chemical composition and processing conditions. One ingredient—the autoclaved whole soybean—served as a control without fermentation. The others were autoclaved whole soybeans or soybean meals fermented with *L. lactis* and then dried at temperatures to deactivate or maintain *L. lactis.* As expected, the defatting and dehulling processes that are done for soybean meal increased AA concentrations when compared with the whole soybean-based ingredients. Composition is only one aspect, however, as protein quality also depends on AA digestibility. In the current study, all indispensable AA digestibilities for fermented protein sources were >80%, with the exception of histidine, valine, and lysine for ASB. That ingredient contained a higher trypsin inhibitor concentration that may have reduced AA digestibility. The indispensable AA digestibilities of the fermented soybean-based ingredients tested in the current study were similar to the ileal AA digestibilities of soybean meal, soy flour, soy protein concentrates, and poultry meal (71.6% to 94.3%) in dogs (Clapper et al., 2001) and ileal AA digestibilities of soybean meal, soy protein isolate, fermented soybean meal, and enzyme-treated soybean meal, fish meal, and casein (77.0% to 99.2%) in weanling pigs (Cervantes-Pahm and Stein, 2010). Collectively, these findings indicate that fermented soybean-based ingredients are comparable to traditional animal, marine, or milk protein sources and may or may not be comparable to non-fermented soy products.

The DIAAS calculations used in this study and other recent studies using the cecectomized rooster model have been a point of discussion in regard to what reference proteins are most appropriate. When DIAAS was originally developed (Food and Agriculture Organization (FAO), 2013), the reference proteins were based on minimum AA requirements. That approach was selected because minimum requirements represent the lowest nutrient concentrations necessary to meet the metabolic and physiological demands of the species in question. Unfortunately, the minimum requirements of some indispensable AA are not known for every life stage (for example, growth, maintenance, gestation, lactation) of dogs and cats. To address this gap in knowledge, an alternative method has been used by our laboratory and others. Instead of NRC minimum requirements alone, the NRC recommended allowances have been used as a reference protein (Reilly et al., 2020a, 2020b; Oba et al., 2023; Smola et al., 2023, 2025). Because the indispensable AA:CP proportions are the same for NRC minimum requirements and NRC recommended allowances (2006), the DIAAS-like scores that result from them are also the same. Additionally, studies on canine and feline AA requirements suggest that the National Research Council (NRC) (2006) recommendations underestimate the physiological needs of these animals (Sutherland et al., 2020; Pezzali et al., 2024). While minimum requirements are theoretically the most appropriate values for DIAAS-like calculations, further research is necessary to validate current recommendations and establish AA requirements across all life stages. Until that research is completed, calculations using either NRC minimum requirements or recommended allowances will yield the same results.

In conclusion, the current experiment demonstrated moderately high AA digestibility values for fermented soybean-based ingredients intended for use in dogs and cat foods. Amino acid digestibilities were typically highest for fermented soybeans + *L. lactis*, likely due to the lower levels of trypsin inhibitor, corresponding to the highest quality protein source. All ingredients performed adequately, with all indispensable AA digestibilities being >80% except for histidine, lysine, and valine in autoclaved soybeans. Based on the DIAAS-like calculations and reference patterns used, methionine + cystine was the first limiting AA for adult dogs, growing puppies, and growing kittens, while phenylalanine + tyrosine was first limiting for adult cats. Our findings indicate that fermented soybean-based ingredients have moderately high AA digestibilities and may serve as adequate proteins in pet foods. Studies evaluating the reproducibility of the fermentation process of the soybean-based ingredients and their effects on diet palatability, nutrient digestibility, and other physiological responses in the target species is suggested.

## Abbreviations:

AA: amino acid
ASB: autoclaved soybeans
CP: crude protein
DIAAS: digestible indispensable amino acid score
DM: dry matter
FSB: fermented soybeans
FSBM: fermented soybean meal
FSBMP: fermented soybean meal + *Lactococcus lactis subsp. lactis* ATCC 11454
FSBP: fermented soybeans + *­Lactococcus lactis subsp. lactis* ATCC 11454
NRC: National Research Council
OM: organic matter
